# The impact of the Drug Marketing Authorization Holder system on sustainable improvement of innovation quality in the pharmaceutical manufacturing industry in China—An empirical study using synthetic control methods

**DOI:** 10.1371/journal.pone.0304056

**Published:** 2024-07-05

**Authors:** Qiang Liu, Su Wang, Zhe Huang

**Affiliations:** 1 School of Business Administration, Shenyang Pharmaceutical University, Shenyang, Liaoning, China; 2 Research Institute of Drug Regulatory Science, Shenyang Pharmaceutical University, Shenyang, China; Inner Mongolia University, CHINA

## Abstract

**Background:**

In November 2015, China launched a pilot program for its Drug Marketing Authorization Holder (MAH) system, aiming to integrate production and research and development resources to spur drug innovation. While many scholars have examined the theoretical relationship between the MAH system and pharmaceutical manufacturing innovation, empirical studies have been scarce.

**Methods:**

This study uses inter-provincial panel data on China’s pharmaceutical manufacturing industry from 2009 to 2019, along with synthetic control methods, to offer a robust analysis of the impact of the MAH paradigm on innovation quality in pilot provinces. We treat the 2015 MAH system implementation in ten provinces as a natural experiment and account for the mediating effects of R&D investments, allowing us to discern the underlying mechanisms.

**Results:**

Our findings demonstrate a significant positive effect of China’s MAH system on innovation quality in the pharmaceutical manufacturing industry. Specifically, the system is most effective in Jiangsu, Guangdong, and Shandong provinces. We also identify R&D investments as playing a mediating role in facilitating the favorable impact of the MAH system on innovation quality.

**Conclusion:**

Our study highlights the crucial importance of the MAH system in promoting innovation quality in China’s pharmaceutical manufacturing industry, providing vital empirical support for its adoption and further enhancement. This research also offers practical insights for policymakers and R&D decision-makers in the pharmaceutical sector.

## 1. Introduction

The Marketing Authorization Holder (MAH) system, a type of drug regulation, has been implemented for many years in developed regions such as Europe, the United States, and Japan. The concept of a MAH was first introduced in the European Economic Community (EEC) Council’s Act 65/65/EEC, which established provisions for drug marketing authorization in the European Union, thereby creating a widely used international MAH system [1]. In the early 20th century, the FDA established its drug registration system, but the federal Food, Drug, and Cosmetic Act (FD&C Act) does not use the term "marketing authorization holder." Instead, "applicant" and "applicant holder" are used to refer to those seeking authorization to market a drug, with "applicant holder" sharing similar obligations as the EU’s MAH [2, 3]. Japan adopted the MAH system following a 2004 amendment to the Pharmaceutical Affairs Law, becoming the first Asian country to do so [4]. China has also formally implemented the MAH system in December 2019.

Currently, China’s economic development is undergoing a shift from high-speed growth to high-quality development [5]. As innovation remains the most significant driving force for economic growth, promoting high-quality innovation activities is essential for achieving high-quality economic development. The pharmaceutical manufacturing industry stands as a strategic pillar industry in ensuring both national security and citizens’ livelihood, making it crucial to achieve sustainable and healthy development. However, more relevant theoretical research is needed. Therefore, the research objective of this paper is to apply the synthetic control method, consider the pilot of the MAH system carried out in some provinces and cities in China from 2015 to 2019 as a quasi-natural experiment, empirically analyze the impact of the MAH system on the quality of innovation of China’s pharmaceutical manufacturing industry and the mechanism of its action, to make up for the deficiencies in the academic research, and to provide the enhancement of the quality of innovation of the pharmaceutical manufacturing industry through the implementation of the MAH system by promoting the Theoretical guidance.

The main objective and innovation of this paper is: Firstly, unlike previous studies, we provide the first empirical analysis exploring the links between the MAH system and the innovation quality of the pharmaceutical manufacturing industry. As such, this paper represents a significant step towards establishing an empirical foundation for the MAH system’s ability to improve the innovation quality in the industry. Secondly, our use of the synthetic control method is noteworthy for its data-driven weighting of the control group, which enables us to obtain a counterfactual control group that closely resembles the treatment group. As a result, this approach minimizes the impact of subjective biases and endogeneity, in contrast to methods such as DID or PSM-DID. Thirdly, alongside assessing the policy’s overall effect, we also examine in-depth the mechanism through which the MAH system can impact the quality of innovation in the pharmaceutical manufacturing industry. Overall, these findings provide valuable insights into the MAH system’s potential to enhance innovation quality in the industry, with wider implications for policymakers and practitioners.

To fulfil these objectives, the study is structured as follows. The first part introduces the basic information of the MAH system and reviews the related research literature. The second part introduces the theoretical foundation of this article and presents the research hypotheses. The third part describes the overall research design ideas, including an introduction to the empirical research methodology and the logic of model setting, data sources, and variable selection. Next, we conducted the empirical analysis, reported the results and conducted validity tests. The fourth part was the mediation effect test. The last part summarizes the conclusions and insights gained from this study and discusses the significance of the study, its limitations and future research directions.

## 2. Institutional background and literature review

Before 2016, China’s drug marketing license system implemented a unified management system that combined the "production license" and the "marketing license" for drugs, i.e., only enterprises holding a "drug production license" could apply for registration, production and sales of drugs. With the rapid development of the economy and the continuous improvement of the industrial system, China’s pharmaceutical manufacturing industry further upgraded the development of this drug production license and marketing license bundled regulatory system, at that time led to a lot of chaos in China’s pharmaceutical manufacturing industry, such as the lack of incentive for research and development of new drugs, a large number of drugs idle approval number, the responsibility for the quality of the drug is not clear and other issues, resulting in the pharmaceutical manufacturing industry as a whole redundant production capacity, the drug supervision departments review and approval resources are wasted, and it is also difficult to protect the legitimate rights and interests of citizens, legal persons and other organizations to participate in the research and development of new drugs at the legal level, which seriously restricts the high-quality development of China’s pharmaceutical manufacturing industry, especially the high-quality development of drug research, development and innovation [6, 7].

To solve the above problems, in August 2015, the State Council formally put forward for the first time in the "Opinions on the Reform of the Review and Approval System for Drugs and Medical Devices" the requirement of "carrying out the pilot of the system of drug listing license holders", and explicitly proposed that "drug research and development institutions and scientific researchers are allowed to apply for registration of new drugs" [8]. and explicitly proposed to "allow drug research and development organizations and researchers to apply for registration of new drugs" [8]. In November of the same year, the Standing Committee of the National People’s Congress authorized the State Council to carry out the pilot system of drug listing permit holders in 10 provinces/municipalities directly under the central government, and the pilot period started from November 5, 2015 to November 4, 2018, with an implementation period of three years; the pilot period was extended for one year on October 26, 2018; and the new version of the Drug Administration Law of the People’s Republic of China was officially released and implemented on December 1, 2019 [9]. On December 1, 2019, the new version of the Drug Administration Law of the People’s Republic of China was formally released and implemented, clearly stating that "the holder of the marketing authorization for drugs shall, in accordance with the provisions of this law, assume responsibility for the non-clinical research, clinical trials, production and operation, post-market research, monitoring of adverse reactions, as well as the reporting and handling of drugs" [9]. At this point, the MAH system was formally established in China through the law.

The existing research literature on the MAH system mainly focuses on the comparative study of the MAH system among countries and the study of the implementation effect of the system. For example, Zhu Jiaxian et al. compared the differences between the marketing authorization holder systems implemented in the European Union, the United States, and Japan, analyzed the regulatory requirements of the MAH system in each country before and after marketing approval, and put forward reference suggestions for improving China’s MAH system [10]. Xie Jinping et al. sorted out the supporting regulatory systems in the whole life cycle management of drugs closely related to the MAH system analyzed the impact on the above regulatory systems before and after implementing the MAH system and put forward targeted suggestions for convergence [11]. Wu Xiaoyan and Huang Zhe analyzed the problems of the existing quality system of domestic commissioned manufacturers after implementing the MAH system. They provided suggestions for the improvement of the quality system of commissioned manufacturers [12]. Jia Jinsheng et al. focused on the evaluation index system of the quality of pharmacovigilance work carried out by the production body after the implementation of the MAH system and explored the design of an informatization platform for pharmacovigilance assessment of marketing license holders, which provides a reference for exploring the intelligence of pharmacovigilance assessment of the holders [13].

However, after literature research and screening, it is found that there needs to be more research on the impact of the implementation of the MAH system on the overall innovation quality of the pharmaceutical manufacturing industry.

## 3. Theoretical foundation and research hypothesis

Considering the implementation experience of countries such as Europe, the United States, and Japan, the core regulatory objective of the MAH system is to realize the separation of the production license from the marketing authorization. It means that MAHs—i.e. holders of marketing authorization documents for drugs, who may be drug manufacturers, R&D institutions, or researchers—are given more autonomy, and they can choose to produce drugs on their own or entrust other manufacturers to carry out production. Through a well-designed institutional framework and implementation pathway, the MAH system revolves around the two core dimensions of rights and responsibilities, aiming to help the regulatory authorities implement management more effectively, thereby effectively preventing risks to drug quality and safety while stimulating the initiative and flexibility of the market’s resource allocation, promoting technology transfer, and incentivizing research and development (R&D) innovation.

According to scholarly consensus, the implementation of the MAH system has the potential to optimize resource allocation within the pharmaceutical manufacturing industry. This would result in more efficient transformation of scientific research outcomes [14–16] and heightened innovation fervor among businesses. Given the high levels of capital investment, long innovation cycles, and frequent uncertainties associated with this field, the externalities of innovation activities have significant sway, particularly within pharmaceutical manufacturing.

In China, the pharmaceutical sector is of strategic importance, with close ties to the nation’s economy and welfare. Therefore, the externalities of innovation activities within the sector mostly arise from national policies. Taken as a whole, the introduction of the MAH system represents a promising development, pointing towards enhanced innovation capabilities and a more robust economic trajectory for China.

The new institutional economic theory presents a fresh approach to analyzing social and economic issues, particularly during the transition between old and new systems [17]. According to this theory, the aim of each system design is to constrain individual behavior that might otherwise maximize welfare or utility by establishing "game rules." At the same time, system design has profound efficiency impacts [18], fostering economic growth via institutional innovation even without increased input factors [19]. It should be noted that not all institutions have historically contributed to economic growth since some institutional arrangements were inefficient or even ineffective. This is because optimal resource allocation is closely tied to the incentive structure of economic society, which itself is determined by the formal system of the government’s supply. Therefore, for the government’s intervention in market operations to promote economic development, it must do so on the precondition of not impinging on the market’s basic regulatory role [19]. The implementation of the MAH system in China aims to transform the previous "drug production and marketing combined management" system into a novel one. The purpose of the MAH system’s implementation is to create more collaborations for the industry, encourage the involvement of more stakeholders in the development of new drugs, as well as internalize various external elements during the new drug research, development, and production process [20]. Additionally, from the standpoint of signal theory, the implementation of the MAH system itself will communicate a signal of the state’s intention to "encourage and innovate in the pharmaceutical manufacturing sector." This signal boosts corporate confidence and enhances their research, development, and innovation capabilities. Consequently, it promotes the entire industry’s high-quality innovation development.

The development of innovative drugs is a complex and multi-faceted process that relies heavily on advancements in basic science and significant investment in research and development. Increasing R&D investment can help companies acquire advanced technologies and high-end equipment, establishing a robust R&D infrastructure while recruiting top scientific talent to enhance innovation capabilities and overall competitiveness. However, the "aggregation effect" of capital allows enterprises to pool resources to tackle cutting-edge technical issues, leading to the production of quality results [21]. While the implementation of the MAH system seems only to relax restrictions on drug production and listing, it also benefits small and medium-sized research institutions, university labs, and other companies lacking large-scale production capacity by allowing them to retain ownership of their innovations after listing and avoiding "invisible" holding [22]. The MAH system also broadens the circulation channels for pharmaceutical industry knowledge assets, attracting private investment in innovative drug R&D and sharing the financial burden of new drug research and development with companies and institutions. This approach allows innovative companies and research institutions to concentrate on developing innovative drugs, dispersing financial risk and enhancing innovation potential [23]. Through policy guidance and market regulation, R&D companies and institutions can achieve relatively equivalent returns from their investments and early-stage risks, enhancing the "liquidity" of innovative drug technologies and stimulating the innovation potential of China’s pharmaceutical manufacturing industry, thus improving overall innovation quality.

Drawing on this information, this paper puts forth the following thesis:

**H1**:The implementation of the MAH system has the potential to enhance the quality of innovation within China’s pharmaceutical manufacturing industry.
**H2**:The adoption of the MAH system enhances the quality of innovation in China’s pharmaceutical manufacturing industry by bolstering the intensity of capital investment in research and development.

**Fig 1:**
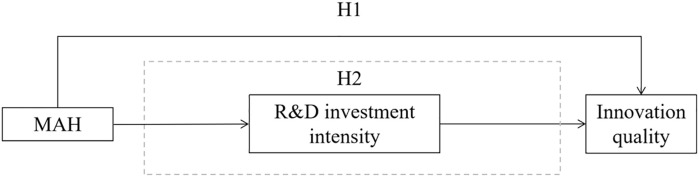
Theoretical framework diagram.

The theoretical model hypothesized in this paper is depicted in Fig 1:

## 4. Materials and methods

### 4.1 Research methods and modeling

This paper adopts the Synthetic Control Method (SCM), which was first proposed by Abadie and Gardeazabal [24]. In the context of this study, the MAH system, which has been piloted in 10 Chinese provinces since 2015, is considered a quasi-natural experiment. Based on this, an experimental group and a control group are established to evaluate the impact of the MAH system on the innovation quality of the Chinese pharmaceutical manufacturing industry. The SCM is a data-driven, non-parametric research method that overcomes problems associated with subjective sample selection and endogeneity that have been problematic for traditional regression methods. As a result, it has been widely employed in research investigating policy implementation effect evaluations in a broad range of fields. For example, evaluations of California’s tobacco control law’s impact [25], the consequences of administrative division adjustments on economic development [26], the impact of trade liberalization on child mortality in emerging and developing countries [27], the impact of China’s carbon emission trading pilot policy on the environment and economy [28], the effects of grassroots environmental protection institutions establishment in China on the environment and economy [29], the impact of developing environmental protection institutions in China, and the impact of the one-child policy on the Chinese economy [30]. This method has also been widely applied in the technological innovation research field. For instance, this paper uses provincial panel data to research the impact of China’s carbon emission trading pilot policy on industrial green technology innovation [31] and how environmental policy affects enterprise innovation ability [32].

Abadie et al. (2015) pointed out in their study that the application of synthetic control should exclude units that are susceptible to policy interventions or specific shocks from the control group. Guided by this idea, the primary operational steps of the synthetic control method used in this paper are as follows: (1) Identifying the appropriate control variables and allocating dissimilar weights for the control group samples; (2) Based on the diverse predictor variable weights, applying the synthetic control method to fit a counterfactual province with comparable characteristics to each MAH system pilot province (i.e., processing group province); (3) Comparing the differences in innovation quality between MAH system pilot provinces and corresponding counterfactual provinces to evaluate the MAH system’s policy effect.

It is assumed that innovation quality data for K+1 (in this paper, K+1 = 20) provinces during the T period can be observed. The first province is affected by the pilot policy in year T_0_ (1≤T_0_≤T), in other words, the processing group provinces, and the remaining K provinces represent the control group provinces. The innovation quality of the pharmaceutical manufacturing industry in province i during t years is referred to as $Y_{it}^{After}$ when the MAH system is implemented, while it is referred to as $Y_{it}^{Before}$ otherwise. Model $Y_{it}=Y_{it}^{Before}+D_{it}\alpha_{it}$ is constructed, where *D*_*it*_ is a dummy variable that indicates whether the MAH policy has been implemented or not. If a particular province i is in the pilot scope for the t period, the variable is valued as 1; otherwise, it is 0. Thus, for all non-pilot provinces, $Y_{it}=Y_{it}^{Before}$ holds throughout the entire T period. The aim of this study was to estimate the value of *α*_*it*_ and the equation is as follows:

$$\alpha_{it}=Y_{it}^{After}-Y_{it}^{Before}=Y_{it}-Y_{it}^{After}$$ (1)

As *Y*_*it*_ represents observable innovation quality data of the pharmaceutical manufacturing industry in the MAH system’s pilot provinces, $Y_{it}^{Before}$ cannot be observed simultaneously in reality. Hence, this paper adopts the factor model proposed by Abadie to construct a "counterfactual" variable from the 19 provinces in the control group:

$$Y_{it}^{Before}=\delta_{t}+\theta_{t}Z_{i}+\lambda_{t}\mu_{i}+\varepsilon_{it}$$ (2)

Formula (1) consists of *δ*_*t*_, which represents the fixed effect over time, *Z*_*i*_, a set of control variables in dimension(r × 1), that remain unaffected by the implementation of the MAH system, and *μ*_*i*_, representing unobserved individual fixed effects in dimension(F × 1). Additionally, *λ*_*t*_ denotes the(1 × F)-dimensional unobservable common factor, while *ε*_*it*_ indicates the error term, whose mean is 0. The method of solving $Y_{it}^{Before}$ involves simulating the characteristics of the treatment group through weighting the control group region. Consequently, by using the set of control variables, a (K + 1) weight vector $W^{*}=\left(w_{2}^{*},\cdots w_{k+1}^{*}\right)$ is computed such that for any k, *w*_*k*_ ≥ 0, and *w*_2_ + ⋯ + *w*_*k*+1_ = 1, it holds. For MAH system pilot areas, vector W represents potential composite control combinations. Moreover, *w*_*k*_ in each combination represents the composite control contribution weight of the provinces in the control group to each province in the processing group, i.e.:

$$\sum_{k=2}^{K+1}\;w_{k}Y_{it}=\delta_{t}+\theta_{t}\;\sum_{k=2}^{K+1}\;w_{k}Z_{k}+\lambda_{t}\;\sum_{k=2}^{K+1}\;w_{k}\mu_{k}+\;\sum_{k=2}^{K+1}\;w_{k}\varepsilon_{kt}$$ (3)

Assuming the existence of an optimal solution vector $W^{*}=\left(w_{2}^{*},\cdots w_{k+1}^{*}\right)$, such that

$$\sum_{k=2}^{K+1}\;w_{k}^{*}Y_{k1}=Y_{11},\cdots \;\sum_{k=2}^{K+1}\;w_{k}^{*}Y_{kT_{0}}=Y_{1T_{0}},\;\text{and}\;\sum_{k=2}^{K+1}\;w_{k}^{*}Z_{k}=Z_{1}$$ (4)

Suppose $\sum_{t=1}^{T_{0}}\;\lambda_{t}^{T}\lambda_{t}$ represents a different matrix; in such a case, there exists:

$$Y_{jt}^{before}-\sum_{k=2}^{K+1}\;w_{k}^{*}Y_{kt}=\sum_{k=2}^{K+1}\;w_{k}^{*}\;\sum_{s=1}^{T_{0}}\;\lambda_{t}{\left(\sum_{i=1}^{T_{0}}\;\lambda_{i}^{T}\lambda_{i}\right)}^{-1}\lambda_{s}^{T}\left(\varepsilon_{ks}-\varepsilon_{1s}\right)-\sum_{k=2}^{K+1}\;w_{k}^{*}\left(\varepsilon_{kt}-\varepsilon_{1t}\right)$$ (5)

Abadie et al. have demonstrated in their research that goal programming can yield an optimal solution, which minimizes the left-hand side of Eq (4). This implies that, during the pilot period, $\sum_{\text{k}=2}^{\text{K}+1}\;{\text{w}}_{\text{k}}^{*}{\text{Y}}_{\text{kt}}$ can be used as a roughly unbiased estimation result for ${\text{Y}}_{\text{jt}}^{\text{before}}$. Thus, when combined with Eq (1), the estimated impact of the MAH system pilot on pharmaceutical manufacturing innovation quality in each province is as follows:

$$\alpha_{it}=Y_{it}^{After}-\sum_{k=2}^{K+1}\;w_{k}^{*}Y_{kt}$$ (6)

The innovation quality of the synthetic province’s pharmaceutical manufacturing industry, obtained through weighting, effectively mirrors the innovation quality development level of the pilot province that has not yet implemented the MAH system, known as the "counterfactual". The disparity in innovation quality between the pilot province and its corresponding synthetic province represents the net impact of the MAH system on the province’s pharmaceutical manufacturing industry innovation quality following its implementation.

### 4.2 Variables

**(1) Dependent variable: Innovation quality(IQ).** Most scholars approach measuring innovation quality from the perspective of patents. For example, Lanjouw and Schankerman [33] employed patent quality as an indicator of innovation quality. They gathered relevant metrics, such as forward and backward citations, to form an index representing patent quality. Zhang Gupeng et al. [34] measured innovation quality using patent grant rate and patent length. As corporations are the primary drivers of innovation, micro-data, such as patent codes and length, are challenging to obtain at a macro level. Therefore, following the practices of Yang Bo et al. [35] and Ma Yonghong et al. [36], this study uses the natural logarithm of the average number of invention patent applications per enterprise in the pharmaceutical manufacturing industry to represent innovation quality (IQ) within a given region.

**(2) Mediating variable.** As per the aforementioned theoretical analysis, the intermediary variable in this paper is R&D capital investment (RDI). The intensity of R&D capital investment within the pharmaceutical manufacturing industry serves as the measurement index.

**(3) Control variables.** To enhance the fitting effect and result robustness of the synthetic control object, it is essential to include critical factors that may impact the innovation quality of the pharmaceutical manufacturing industry as control variables, as per the practice of Liu Jiayan and Fan Ziying [37]. Drawing from previous studies [37–40], this paper identifies seven variables that may influence the industry’s innovation quality, which are subsequently added as control variables. Table 1 illustrates the variable names and their respective definitions as used in this paper.

**Table 1:** Variable definitions.

Variable type	Variable name	Variable symbol	Description
Dependent variable	Innovation quality	IQ	Ln(Number of invention patent applications/enterprises)
Mediating variable	R&D investment	RDI	Internal expenditure of R&D funds/main business income
Control variables	Cooperative R&D investment	CRI	R&D external expenditure/main business income
	economic development level	Eco	Ln(GDP Per Capita)
	Industry profitability	Prof	Ln(total profit)
	government support	Gov	Ln(The amount of government funds in the internal R&D expenditure)
	industrial scale	Scale	Ln(Average number of employees)
	market competition	Market	Ln(number of the enterprises)
	capital-intensity	Capin	Ln(Capital stock of assets per capita)

To eliminate the impacts of price factors, this paper deflates the main business income using the producer price index (PPI) published by the National Bureau of Statistics (2009 = 100); the total profit is deflated using the consumer price index (CPI) published by the same bureau (2009 = 100); while the total assets are deflated using the fixed asset investment index also published by the National Bureau of Statistics (2009 = 100). Meanwhile, Zhu Pingfang et al.’s method [41] for constant price treatment is adopted for the R&D internal expenditure price index, whereby a fixed investment price index and consumer price index are attributed 0.45 and 0.55, respectively.

Table 2 displays the descriptive statistical results for each variable.

**Table 2:** Descriptive statistics results.

Variable	N	Mean	SD	Min	Max
IQ	319	1.390	1.602	0.091	14.000
RDI	319	0.018	0.009	0.001	0.044
RDP	319	7.680	1.204	1.740	9.830
CRI	318	0.003	0.003	0.000	0.025
Eco	319	10.710	0.499	9.241	12.010
Prof	314	3.856	1.226	-2.810	6.187
Gov	319	8.114	1.295	3.091	10.760
Scale	319	10.780	0.931	8.082	12.480
Market	319	5.157	0.911	2.197	6.755
Capin	319	4.585	0.496	2.179	5.648

### 4.3 Sample selection and data

This paper utilizes panel data from 29 provinces in China’s pharmaceutical manufacturing industry spanning from 2009 to 2019 as research samples. Qinghai and Tibet were excluded due to extensive missing data. Experimental subjects were selected from ten provinces participating in the pilot, namely Beijing, Shanghai, Tianjin, Hebei, Shandong, Jiangsu, Zhejiang, Fujian, Guangdong, and Sichuan. Meanwhile, 19 provinces outside the pilot area were chosen as the control group. The selected research interval holds significance for two reasons: firstly, data on the number of invention patent applications of the pharmaceutical manufacturing industry in various provinces before 2009 were missing, and secondly, the MAH system pilot concluded on December 1, 2019, after which it was rolled out countrywide. All the data utilized in this paper were sourced from the China High-tech Industry Statistical Yearbook and provincial statistical yearbook throughout the years.

## 5. Empirical results and analysis

### 5.1 Baseline empirical results

Although the MAH system pilot was launched simultaneously in ten provinces, the heterogeneity in economic development, geographical conditions, and industrial structure among these provinces would inevitably result in variation in the innovative quality of the pharmaceutical manufacturing industry. However, such variability does not pose a threat to the robustness of the empirical analysis results presented in this paper. The synthetic control method is a data-driven empirical analysis tool that permits the examination of the innovation quality of pharmaceutical manufacturing industries in provinces irrespective of their innovation quality levels. As long as synthetic control provinces manifest a better fit to the changing trend of innovation quality in pilot provinces before policy implementation, the effect of policy implementation can be effectively reflected [42]. Comprehensively utilizing the synth command written by Abadie et al and the synth2 command written by Professor Chen Qiang’s team of Shandong University, corresponding synthetic control provinces for each pilot province in Stata17.0 software were constructed, and the weights for each synthetic control province were computed as indicated in Table 3. The greater the weight assigned to a particular composite province, the more similar its characteristics are to the corresponding pilot province. A weight of 0, on the other hand, indicates significantly varied features between the composite and pilot provinces. For instance, the synthetic control provinces and their corresponding weights for Zhejiang Province are Yunnan (0.451), Hubei (0.425), Chongqing (0.091), and Heilongjiang (0.033), with all other provinces assigned a weight of 0, resulting in a total weight of 1. This demonstrates that the innovation quality index of the pharmaceutical manufacturing industry in Yunnan, Hubei, Chongqing, and Heilongjiang can be estimated based on their weights of 0.451, 0.425, 0.091, and 0.033, allowing for the estimation of the developmental trend of innovation quality in Zhejiang Province without the need for implementing the MAH system pilot.

**Table 3:** Weights of synthetic control provinces corresponding to MAH system pilot provinces.

Pilot province	Zhejiang	Tianjin	Sichuan	Shanghai	Shandong	Jiangsu	Hebei	Guangdong	Fujian	Beijing
Synthesize provinces and weights	Yunnan(0.451)	Hainan(0.900)	Jiangxi(0.650)	Heilongjiang(0.403)	Guizhou(0.339)	Heilongjiang(0.536)	Henan(0.612)	Heilongjiang(0.659)	Gansu(0.308)	Chongqing(0.430)
	Hubei(0.425)	Chongqing(0.100)	Guizhou(0.350)	Guizhou(0.242)	Jiangxi(0.333)	Guizhou(0.275)	Chongqing(0.288)	Guizhou(0.291)	Jiangxi(0.257)	Inner Mongolia(0.188)
	Chongqing(0.091)			Liaoning(0.178)	Shanxi(0.176)	Jilin(0.114)	Gansu(0.100)	Hainan(0.050)	Guizhou(0.215)	Shanxi(0.187)
	Heilongjiang(0.033)			Chongqing(0.104)	Chongqing(0.144)	Henan(0.074)			Liaoning(0.103)	Hunan(0.106)
				Hainan(0.074)	Ningxia(0.007)				Inner Mongolia(0.069)	Hainan(0.089)
									Hainan(0.038)	
									Heilongjiang(0.008)	

**Fig 2:**
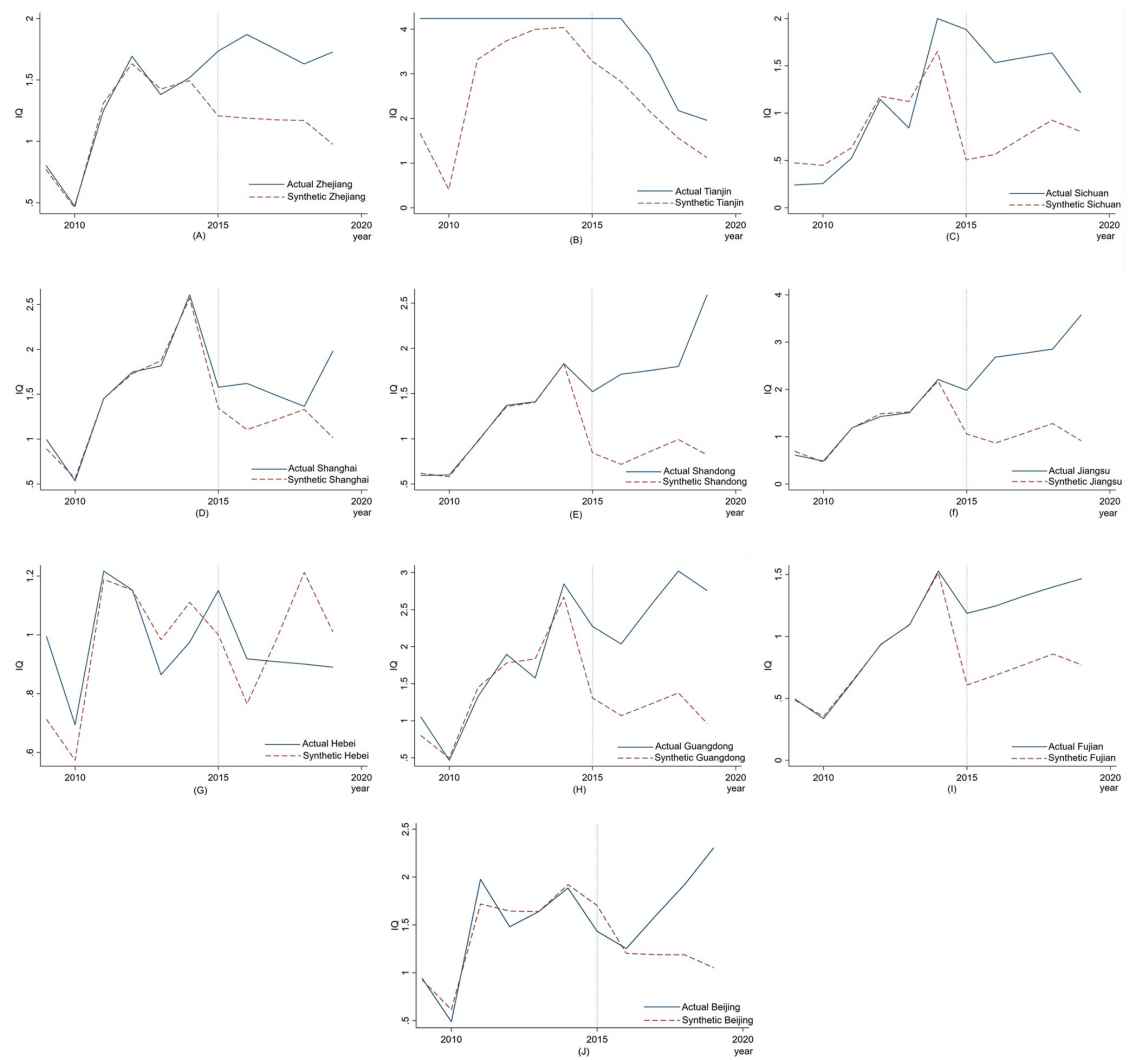
The innovation quality trends of the pharmaceutical manufacturing industry in pilot and synthetic provinces under the MAH system.

Fig 2 shows the evolution trend of pharmaceutical manufacturing innovation quality in 10 MAH system pilot provinces and their corresponding synthetic control provinces. The solid lines depict the actual development trend of pharmaceutical manufacturing innovation quality in the pilot province, while the dotted lines represent the corresponding development trend in the synthetic control province. The vertical dotted line corresponds to the year of the pilot implementation of the MAH system. It is evident that before the vertical dotted line, the real pharmaceutical manufacturing innovation quality of provinces such as Zhejiang (Fig 2A), Shanghai (Fig 2D), Shandong (Fig 2E), Jiangsu (Fig 2F), Guangdong (Fig 2H), Fujian (Fig 2I) and Beijing (Fig 2J) almost coincided with the corresponding synthetic curves of pharmaceutical manufacturing innovation quality development. This implies that the synthetic control method aligns well with the actual development trend of pharmaceutical manufacturing industry innovation quality in these pilot provinces. However, the fitting results in Sichuan (Fig 2C) were poor, and the fitting results in Tianjin (Fig 2B) and Hebei (Fig 2G) were the worst, indicating that the pharmaceutical manufacturing industries in Sichuan, Tianjin and Hebei provinces possessed unique innovation quality characteristics and other related economic characteristics that made it challenging to obtain a perfect fit by computing the weights of other provinces and cities.

Table 4 shows the R-squared values of the fitted synthetic control method for the aforementioned 10 pilot provinces. As can be seen, except for Tianjin, Sichuan and Hebei, the R-squared values of the other seven pilot provinces are close to unity. This indicates that the synthetic control method’s fitting effect is excellent in these seven provinces: Zhejiang, Shanghai, Shandong, Jiangsu, Guangdong, Fujian and Beijing. Therefore, our subsequent analysis will concentrate on these seven provinces only.

**Table 4:** The synthetic control method’s fitting results for each pilot province.

Pilot province	Zhejiang	Tianjin	Sichuan	Shanghai	Shandong	Jiangsu	Hebei	Guangdong	Fujian	Beijing
R-squared	0.989	-1.026	0.736	0.994	0.999	0.994	0.605	0.937	0.999	0.917

As depicted in Fig 2, following the MAH system pilot’s launch, the actual and synthetic pharmaceutical manufacturing innovation quality trends in six provinces, namely Zhejiang, Shanghai, Shandong, Jiangsu, Guangdong and Fujian, gradually diverged. The real change trend line (i.e., the solid line) was above the synthetic change trend line (i.e., the dashed line). In other words, the actual pharmaceutical manufacturing industry innovation quality in these six provinces was higher than the synthetic innovation quality after the pilot implementation of the MAH system, implying a higher innovation quality than what would have been anticipated without the pilot. Although Beijing’s pharmaceutical manufacturing industry innovation quality was slightly lower than that of the first year after the policy implementation, it has exceeded that of Beijing significantly since then. This is likely because Beijing is the political and regulatory center of our country’s pharmaceutical manufacturing industry. As such, the local enterprises and regulatory authorities may exercise greater caution in implementing and promoting the MAH system pilot to minimize the uncertainty risks associated with such innovative policies, as compared to the other pilot provinces.

Moreover, the real innovation quality trend of the pharmaceutical manufacturing industry in each pilot province after the MAH system pilot implementation differs from the innovation quality trend of its synthetic counterpart. Fig 2A shows that the innovation quality of the pharmaceutical manufacturing industry in Zhejiang Province initially fluctuated and declined after the policy implementation, but changed after the MAH policy was introduced. Despite a decline from 2016 to 2018, the industry still exhibited an upward trend thereafter. Contrarily, Fig 2D portrays that the innovation quality of the pharmaceutical manufacturing industry in Shanghai experienced a significant decline from 2014, which persisted until 2018. However, due to the MAH system’s pilot implementation, the downward trend slowed after 2015 and rebounded after 2018, resulting in a steady improvement. In contrast, Fig 2E, 2F and 2I reveal that the innovation quality of the pharmaceutical manufacturing industries in Shandong, Jiangsu, and Fujian continued to grow after the policy implementation. Although the growth trend of Shandong and Jiangsu decelerated slightly from 2016 to 2018, they continued to show a rapid expansion thereafter. As illustrated in Fig 2h, the innovation quality of the pharmaceutical manufacturing industry in Guangdong Province briefly declined within the first year of the policy’s implementation before steadily improving for several years. Despite a brief decline in 2019, the industry still exhibited an overall upward trend. Finally, in Fig 2j, it can be observed that the innovation quality of Beijing’s actual pharmaceutical manufacturing industry briefly declined within the first year after the policy’s implementation before experiencing a lag period of about one year, after which it resumed rapid growth.

To thoroughly examine the effect of the MAH system pilot on the innovative quality of the pharmaceutical manufacturing industry in different provinces, this study quantifies the average innovation quality value in the actual and counterfactual cases of this industry in the seven above-mentioned provinces during the pilot period. The corresponding data is presented in Table 5. According to the table, for the pilot duration of the MAH system, the innovation quality of the pharmaceutical manufacturing industry in Jiangsu, Guangdong, and Shandong provinces ranked among the top three of all seven pilot provinces. Furthermore, the treatment effect values of these three provinces also placed in the top three, indicating that the pilot implementation of the MAH system in these regions had the most significant impact on improving the innovative quality of the respective pharmaceutical manufacturing industries.

**Table 5:** Comparison between the actual and synthetic values of innovation quality in the pharmaceutical manufacturing industry of the MAH system pilot province.

Unit	Mean Actual Outcome	Mean Synthetic Outcome	Mean Treatment Effect
Zhejiang	1.743	1.144	0.599
Shanghai	1.607	1.202	0.404
Shandong	1.876	0.847	1.029
Jiangsu	2.772	1.038	1.734
Guangdong	2.526	1.188	1.338
Fujian	1.325	0.739	0.586
Beijing	1.702	1.267	0.435

## 5.2 Validity checks

**(1) Placebo test.** Based on the empirical analysis presented above, it is clear that there exists a significant disparity between the actual and synthetic innovation qualities of the pharmaceutical manufacturing industry in Zhejiang, Shanghai, Shandong, Jiangsu, Guangdong, Fujian and Beijing due to the pilot implementation of the MAH system. In order to ascertain that this difference is not attributed to random factors, but rather due to the MAH pilot implementation, this study applies the placebo approach, utilizing the methods employed in previously published literature [25, 43, 44], to analyze the policy impact of the MAH system pilot. The placebo test operates under the fundamental notion: select a non-pilot province as a placebo province, and assume that both the placebo province and an actual pilot province underwent the same policy treatment within the same year. Then, using the synthetic control method to conduct identical analysis. This test verifies that if the calculated policy treatment effect of the placebo province is lesser than that of the corresponding actual pilot province, then the innovation quality variation between the synthetic province of pharmaceutical manufacturing industry and the actual pilot province is genuinely a result of the MAH pilot implementation, that is, the empirical analysis results are valid. On the contrary, if the policy treatment effect of the placebo province is greater than that of the corresponding actual pilot province, the empirical analysis outcomes are deemed ineffective.

**Fig 3:**
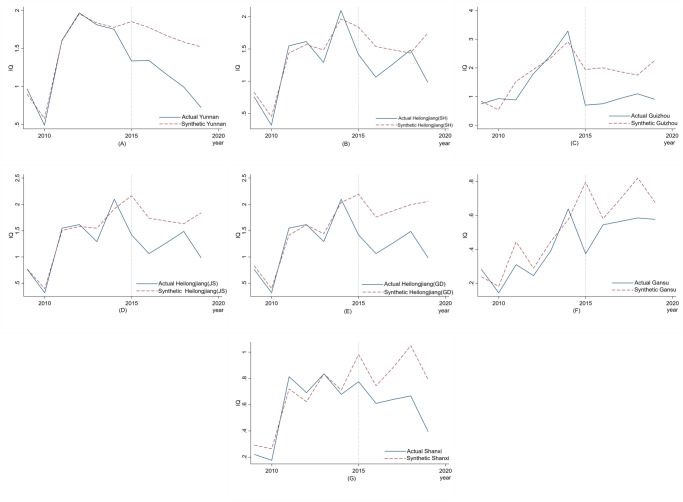
Validity checks——Placebo test.

In this study, the placebo provinces were selected from the control group provinces with the highest similarity to the pilot provinces, based on the synthetic contribution rate derived from fitting the synthetic control method—these provinces are presented in the second row of Table 3. As shown in Fig 3, the corresponding actual and synthetic innovation quality change trend curves of Yunnan, Heilongjiang (SH), Guizhou, Heilongjiang (JS), Heilongjiang (GD), Gansu, Shanxi serve as placebo provinces. The fitting weights of each placebo province are exhibited in Table 6. For Fujian, despite Gansu and Jiangxi ranking as the top two provinces in terms of weight contribution under synthetic control, they demonstrated limited fitting to the actual situation prior to 2015, resulting in R-squared values of 0.72786 and 0.66745 respectively, thus the policy effect cannot be effectively reflected. Therefore, Guizhou, which had the third-highest contribution weight to synthetic Fujian, was selected as the placebo test province. Similarly, for Beijing, Shanxi was chosen as the placebo test province due to the suboptimal fitting effects observed in Chongqing (R^2^ = 0.57767) and Inner Mongolia (R^2^ = 0.51229).

**Table 6:** The fitting results of synthetic control method for each placebo province.

Pilot province	Zhejiang	Shanghai	Shandong	Jiangsu	Guangdong	Fujian	Beijing
Placebo province	Yunnan	Heilongjiang(SH)	Guizhou	Heilongjiang(JS)	Heilongjiang(GD)	Guizhou	Shanxi
R-squared	0.991	0.940	0.819	0.935	0.968	0.845	0.905

Illustrated in Fig 3, with the exception of Heilongjiang, the placebo province of Shanghai showed a slightly lower synthetic path compared to the true path in 2018, while all other placebo provinces had a higher synthetic path. These results suggest that provinces with the highest similarity to the pilot provinces did not experience similar effects after implementation of the MAH system, indicating that the effects observed were not due to random factors, but indeed attributable to the MAH policy.

**(2) Ranking test.** In order to further test the statistical significance of the effect of the MAH pilot policy, a ranking test method, similar to the one proposed by Abadi et al. [25], was used in this study. The specific idea behind this method involves randomly selecting a series of non-pilot provinces from the control group and assuming that they were also affected by the same policy impact in 2015. The synthetic control method is then used to construct the innovation quality results of the pharmaceutical manufacturing industry in each of these selected provinces. Next, a series of random "policy effects" are obtained, which represent the difference between the real and synthetic values of each non-pilot province. The policy effect of the corresponding pilot province is then compared with the policy effect of the randomly selected non-pilot provinces. If the policy effect of the pilot province is significantly greater than that of the randomly selected non-pilot province, it indicates that the effect of the policy on the pilot province is significant. It should be noted that the ranking test method requires a good fitting effect of the provinces in the control group prior to policy implementation. If the fitting effect before policy implementation is poor, it suggests that the final "policy effect" may be due to the poor fitting effect and not related to the policy implementation.

**Fig 4:**
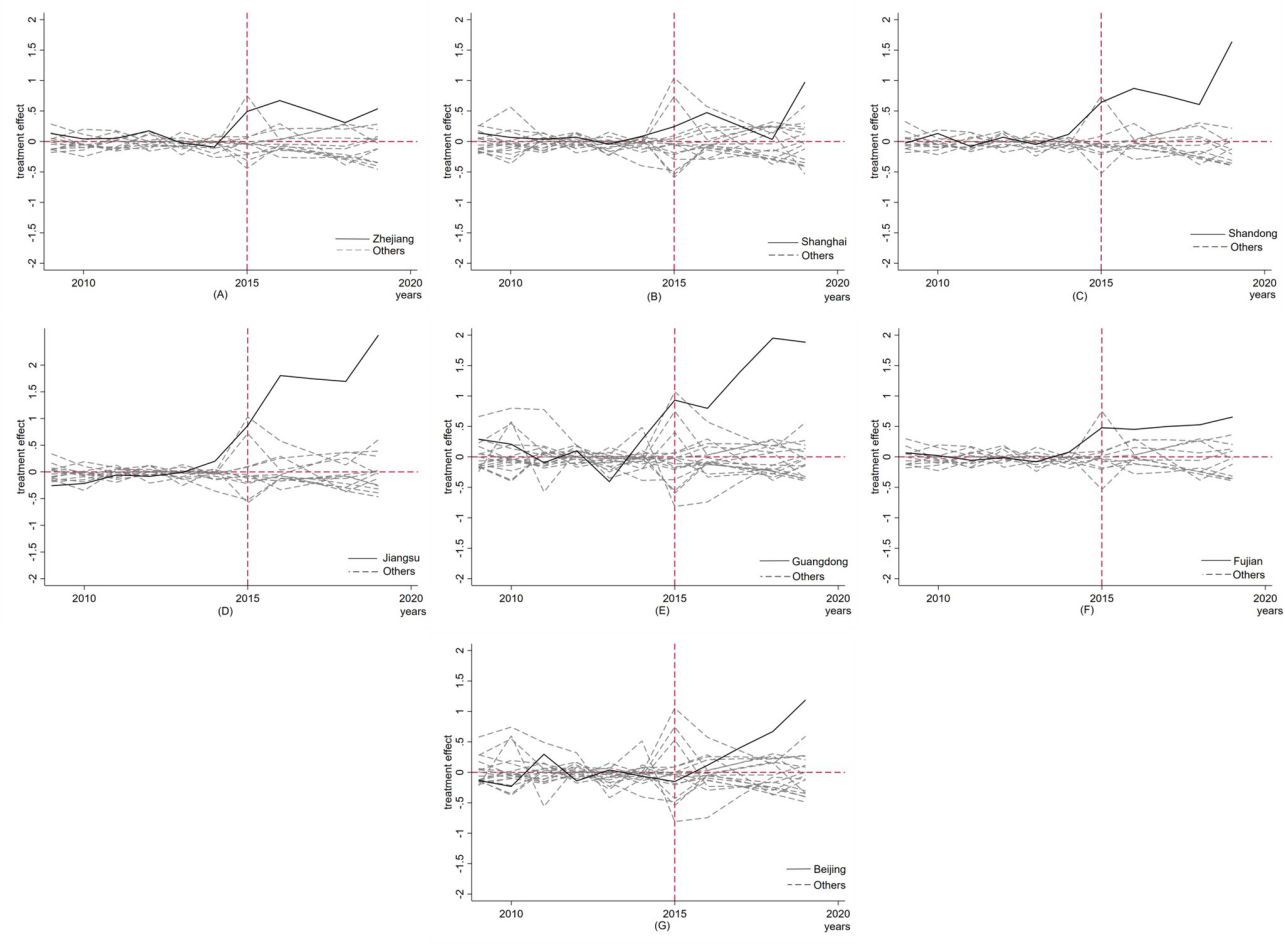
Validity checks——Ranking test.

This study evaluated the fitting effect of the provinces in the control group prior to policy implementation by calculating and comparing their mean square prediction error (MSPE) values. Provinces in the control group whose MSPE values were 20 times greater than the MSPE values of the pilot provinces were excluded, and the remaining provinces were identified as the provinces in the ranking test. Based on this methodology, a line chart of ranking test results was created for each pilot province, as illustrated in Fig 4. The chart shows that the policy processing effect of the 7 pilot provinces was significantly higher than that of the non-pilot provinces in their respective control groups. This implies that if the control group provinces intend to achieve the same policy processing effect as the pilot provinces, it is a low probability event, thereby indicating that the policy effect of the MAH system pilot is significant.

### 5.3 Robustness checks

To address the potential problem of variations in analysis results due to different control group selections, this study adopts the iterative method in accordance with the approach of Su Zhi and Hu Di et al. [45] to conduct a robustness test. For each pilot province, one control group province with a positive contribution towards its synthesis is eliminated iteratively, and the pilot province and the composite province are re-estimated. This methodology aims to examine whether the effect of the MAH system on the innovation quality of the pharmaceutical manufacturing industry in each pilot province will be affected due to the lack of control group provinces.

**Fig 5:**
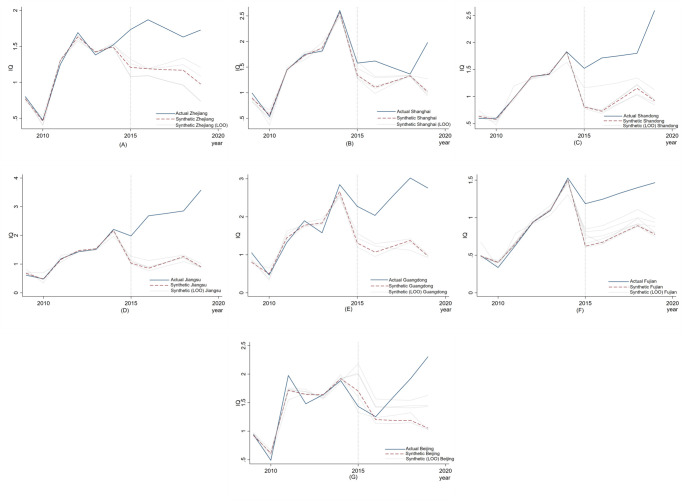
Robustness checks——Iteration method.

The test results are presented in Fig 5.

The results in Fig 5(a)–5(g) demonstrate that after successive changes in the composition of the control group of the 7 pilot provinces, the trend of the broken line is consistent with the fluctuation range and the actual composite provinces. Additionally, the study employed the LOO (leave-one-out) method to quantitatively estimate the results [46]. If the fitting effect of the original synthetic control is not affected by the elimination of some provinces in the control group, the empirical result can be considered robust. On the contrary, if the fitting results were driven by the eliminated synthetic control province, the results would not be robust.

Table 7 summarizes the LOO estimation results. The real results of the experimental group and its Synthetic control results, i.e., Synthetic Outcome, as well as the synthetic control results after iterative elimination using the LOO method, i.e., synthetic Outcome (LOO), are presented. After applying the LOO method to the synthetic control, stata calculates corresponding results for each province in the control group with a positive weight. However, for clarity, stata only reports the maximum and minimum values corresponding to each year.

**Table 7:** Robustness test results.

Unit	Year	Synthetic Outcome		Synthetic Outcome (LOO)	
		Actual	Synthetic	Min	MAX
Zhejiang	2016	1.870	1.189	1.093	1.191
	2017	1.751	1.176	1.023	1.261
	2018	1.630	1.170	0.962	1.341
	2019	1.728	0.976	0.738	1.209
Shanghai	2016	1.619	1.106	0.985	1.321
	2017	1.490	1.215	1.164	1.326
	2018	1.365	1.330	1.321	1.343
	2019	1.981	1.017	0.934	1.273
Shandong	2016	1.714	0.730	0.687	1.204
	2017	1.755	0.940	0.869	1.260
	2018	1.800	1.153	1.029	1.346
	2019	2.592	0.923	0.845	1.124
Jiangsu	2016	2.683	0.853	0.766	1.126
	2017	2.764	1.054	0.931	1.214
	2018	2.853	1.265	1.086	1.315
	2019	3.579	0.908	0.870	1.016
Guangdong	2016	2.036	1.069	0.956	1.295
	2017	2.542	1.220	1.144	1.349
	2018	3.020	1.376	1.119	1.422
	2019	2.756	0.965	0.925	1.013
Fujian	2016	1.246	0.674	0.651	0.895
	2017	1.328	0.786	0.770	0.997
	2018	1.400	0.894	0.886	1.109
	2019	1.466	0.777	0.748	0.984
Beijing	2016	1.254	1.203	1.139	1.564
	2017	1.594	1.189	1.134	1.549
	2018	1.922	1.188	1.149	1.541
	2019	2.306	1.053	1.015	1.628

If the value of the Synthetic control result of the experimental group falls between the maximum value and the minimum value obtained after the elimination of LOO iteration, it implies that the elimination of a control group does not affect the synthetic control fitting results of the original experimental group, and the empirical results are thus robust. All values listed in Table 7 satisfy this requirement, which proves that the empirical results are robust. In addition, the process and results of the above empirical analyses and tests are consistent with the results reported in other literatures [25–32, 43–45] that adopt similar analytical methods, which also proves that the empirical results of this paper are reliable and trustworthy, i.e., the implementation of the MAH system has a significant contribution to the quality of innovation of China’s pharmaceutical manufacturing industry, and H1 has been verified up to this point.

## 6. Further analysis

The above empirical research results demonstrate that the MAH system significantly improves the innovation quality of the pharmaceutical manufacturing industry in pilot provinces. However, the underlying mechanism for this improvement remains to be explored. According to the theoretical analysis described above, the pilot MAH system has the potential to enhance the intensity of R&D capital investment (H1) of enterprises, which can affect the innovation quality of the pharmaceutical manufacturing industry. To test whether this mechanism is valid, empirical evidence will be presented in this section.

### 6.1 Model specification

Drawing on the research practices of Zhou Zhou [38], Wen Zhonglin, Ye Baojuan [47], and others, we have constructed the following model using the chain mediation effect analysis method to test our hypothesis:

$${\text{InnoQT}}_{\text{it}}=\alpha_{\text{it}}+\beta_{1}{\text{treat}}_{\text{it}}+{\delta \text{control}}_{\text{it}}+\varepsilon_{\text{it}}$$ (7)

$${\text{M}}_{\text{it}}=\alpha_{\text{it}}+\beta_{2}{\text{treat}}_{\text{it}}+{\delta \text{control}}_{\text{it}}+\varepsilon_{\text{it}}$$ (8)

$${\text{InnoQT}}_{\text{it}}=\alpha_{\text{it}}+\beta_{3}{\text{M}}_{\text{it}}+{\delta \text{control}}_{\text{it}}+\varepsilon_{\text{it}}$$ (9)

$${\text{InnoQT}}_{\text{it}}=\alpha_{\text{it}}+\beta_{4}{\text{treat}}_{\text{it}}+{\theta \text{M}}_{\text{it}}+{\delta \text{control}}_{\text{it}}+\varepsilon_{\text{it}}$$ (10)

In this model, treat_it_ represents the policy dummy variable of the MAH system pilot, which is derived from the multiplication of a dummy variable that represents the processing group and a dummy variable that represents the time point of policy intervention. Specifically, if province i implements the MAH system pilot at time point t, the value of this variable is 1; otherwise, it is 0. M_it_ is the intermediate variable, which represents research and development investment intensity (RDI) and control_it_ represents the combination of control variables.

### 6.2 Analysis of the mediating effect

Table 8 presents the test results of the mediating effect of the MAH system pilot on promoting the improvement of innovation quality in the pharmaceutical manufacturing industry. Columns (1)—(4) display the regression results of models (7)—(10), respectively. The results in column (1) demonstrate that the coefficient β_1_ of the policy dummy variable (Treat) is significantly positive at the 1% level, indicating that the MAH system has a significant promoting effect on the innovation quality of the pharmaceutical manufacturing industry in the pilot provinces. This aligns with the conclusion obtained by the synthesis control method above and provides the premise for further analysis of the mediating effect.

**Table 8:** Results of the mediation effect test.

VARIABLES	column(1)	column(2)	column(3)	column(4)
	IQ	RDI	IQ	IQ
Treat	0.700***	0.002*		0.568***
	(0.147)	(0.001)		(0.128)
RDI			43.490***	38.150***
			(6.850)	(6.455)
CRI	40.370**	0.470***	25.990	29.270
	(16.240)	(0.170)	(16.020)	(17.860)
Eco	0.021	0.004**	-0.020	-0.132
	(0.211)	(0.002)	(0.207)	(0.203)
Prof	0.002	-2.35e-06	-0.005	-0.009
	(0.043)	(0.001)	(0.042)	(0.039)
Gov	0.034	-6.69e-05	0.031	0.052
	(0.043)	(0.001)	(0.041)	(0.039)
Scale	0.539**	0.005**	0.569***	0.426**
	(0.225)	(0.002)	(0.218)	(0.207)
Market	-0.609***	-0.007***	-0.556**	-0.419**
	(0.222)	(0.002)	(0.217)	(0.208)
Capin	0.499***	0.006***	0.322*	0.308*
	(0.170)	(0.002)	(0.169)	(0.178)
Constant	-4.500**	-0.0732***	-4.511**	-2.617
	(2.279)	(0.024)	(2.212)	(2.263)
Observations	280	280	280	280
Number of city	26	26	26	26

Standard errors in parentheses:

*** p<0.01,

** p<0.05,

* p<0.1

Using research and development investment intensity (RDI) as the mediating variable, the results in column (2) show that the regression coefficient β_2_ of the policy dummy variable (Treat) is significantly positive at the 10% level, implying that the implementation of the MAH system increases R&D investment intensity in pilot provinces. The results in column (3) reveal that the regression coefficient β_3_ of the mediating variable is significantly positive at the 1% level, indicating that the R&D investment intensity significantly affects the improvement of innovation quality, suggesting that the indirect effect of the entire model is significant. The results in column (4) show that the regression coefficient β_4_ of the policy dummy variable (Treat) is still significant, implying that the direct effect is significant. Furthermore, β_2_, β_3_, β_4_ are all positive, indicating that the intensity of R&D capital investment plays a partial mediating effect in the process of improving the innovation quality of the pharmaceutical manufacturing industry by the MAH system, accounting for 12.55% ((0.002*43.490) /0.700) of the total effect. Therefore, the MAH system can enhance the quality of innovation by strengthening the intensity of R&D investment in the pharmaceutical manufacturing industry. Hence, hypothesis H2 is verified.

There are several possible reasons for this finding: First, the implementation of the MAH system enables non-manufacturing enterprises to become the primary applicant for registration and listing of new drugs, establishing a business mechanism of equal investment and benefit, which greatly stimulates the enthusiasm of enterprises to use funds for innovative drug research and development and also promotes the inflow of social and venture capital. Second, the MAH system has promoted the separation of drug research and development and production, which has further released the idle capacity of production enterprises, reduced the operating costs of production enterprises, and can transfer the cost savings to the research and development link. Thus, the MAH system improves the quality of innovation in the pharmaceutical manufacturing industry by enhancing the intensity of investment in research and development funds.

## 7. Conclusion and implications

The Marketing Authorization Holder system is a mature and well-established drug regulatory framework in the world. It plays a crucial role in promoting rational and integrated utilization of production resources and stimulating innovation vitality in the pharmaceutical manufacturing industry. Utilizing panel data from 29 provinces in China between 2009 and 2019, this study employs the quasi-natural experiment approach to analyze the impact of the pilot policies of MAH system implemented in 10 provinces in 2015 on the innovation quality of the pharmaceutical manufacturing industry using the synthetic control method. Based on the empirical analysis, the following conclusions were drawn: (1) The implementation of the MAH system significantly improved the innovation quality of the pharmaceutical manufacturing industry in the pilot provinces, with Jiangsu Province having the most significant improvement effect, followed by Guangdong Province and Shandong Province. (2) R&D capital investment played a significant intermediary role in the process of improving the innovation quality of the pharmaceutical manufacturing industry through the MAH system, highlighting how the MAH system can boost innovation quality by strengthening the R&D capital investment in the pharmaceutical manufacturing industry.

Based on the above conclusions, the following implications can be drawn:

① The Chinese drug regulatory authorities must fully appreciate the pivotal role of the MAH system in advancing innovation quality within the pharmaceutical manufacturing industry. They should clarify its mechanism of action and introduce targeted management rules and incentive measures in accordance with the differentiated development stage of the industry across different provinces. Furthermore, the authorities should leverage effective policy tools to create a robust research environment for MAH, upstream and downstream related enterprises, and ensure that the institutional dividends from the MAH system’s efficacy in enhancing innovation quality are maximized.
② Adequate investments in research and development have played an indispensable role in facilitating MAH’s efforts to promote high-quality innovation in the pharmaceutical manufacturing industry. To this end, both enterprises and government departments must coordinate and work in tandem, leveraging a development strategy that incorporates "market leadership" and "government regulation." On the one hand, government regulators should authorize tax subsidies, research and development project grants, and other forms of financial aid to key innovative enterprises while establishing policies that incentivize pharmaceutical research and development innovation. On the other hand, production enterprises or research and development institutions should effectively allocate their investment portfolios, prioritize internal and external new product research and development projects, maximize the MAH system’s efficacy in augmenting enterprise innovation capacity, and ultimately enhance the overall innovation quality of China’s pharmaceutical manufacturing industry.

This study enriches the academic research on the MAH system and is an essential supplement to the existing research results; it can provide crucial theoretical references for the countries that have already implemented the MAH system and those international countries that are about to implement the MAH system. It also provides important theoretical references for the MAH system’s self-improvement and coordinated development with industrial innovation and socio-economics. Therefore, the research results of this paper will be highly concerning to food and drug regulatory agencies, pharmaceutical research institutes, and enterprises of marketing authorization holders in various countries.

This study still has certain limitations. First, due to the limitations of the data, the sample size included in the empirical study of this paper is small; second, this paper only selects the relevant data in China as the research object, which leads to a certain lack of international generalizability of the results of this paper; third, the mediating effect of the variable of the intensity of R&D capital investment in this paper only accounts for 12.55% of the total effect, which indicates that there are still other mediating variables. Due to data limitations, it is not possible to conduct a comprehensive and in-depth investigation of the mechanism of the MAH system affecting the innovation quality of the pharmaceutical manufacturing industry. Therefore, in the subsequent research, it is necessary tto expand the sample size of empirical analysis further and search for more mediating variables to more comprehensively analyze the path of the MAH system’s influence on the innovation quality of the pharmaceutical manufacturing industry; at the same time, as far as possible, the data of different countries should be selected for empirical analysis to improve the generality and practicability of the conclusions of this study.
